# 2-Amino-5-nitro­pyridinium tetraoxido­rhenate(VII) monohydrate

**DOI:** 10.1107/S1600536809029365

**Published:** 2009-07-29

**Authors:** V. H. Rodrigues, M. M. R. R. Costa, T. Dekola, E. de Matos Gomes

**Affiliations:** a CEMDRX, Department of Physics, University of Coimbra, P-3004-516 Coimbra, Portugal; b Departamento de Física, Universidade do Minho, P-4710-057 Braga, Portugal

## Abstract

All the residues of the title compound, (C_5_H_6_N_3_O_2_)[ReO_4_]·H_2_O, are located on general crystallographic positions. The 2-amino-5-nitro­pyridinium cation has a typical planar conformation with one of the nitro O atoms −0.058 (5) Å out of plane; the amine H atoms are also a little out of the main ring plane towards the opposite side of the aforementioned O atom [by 0.02 (4) and 0.04 (4) Å]. The perrhenate anion is nearly ideally tetra­hedral. Three distinct N—H⋯O hydrogen bonds give rise to *C*(8) zigzag chains running along [100]. *R*_4_^4^(12) rings involving the two hydrogen bonds in which the water mol­ecules inter­act with the perrhenate anions are also present.

## Related literature

For the structural analyses of related 2-amino-5-nitro­pyridium salts and their potential application as non-linear optical materials, see: Masse & Zyss (1991 ▶); Puig-Molina *et al.* (1998 ▶); Aakeröy *et al.* (1998 ▶); Pecaut *et al.* (1993 ▶). 
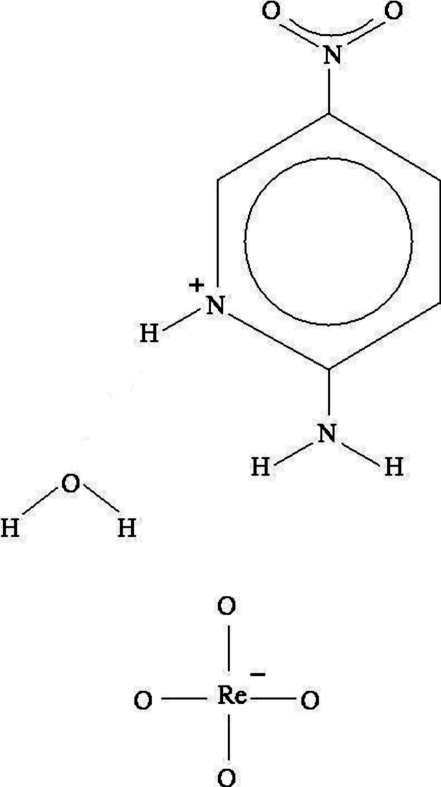


## Experimental

### 

#### Crystal data

(C_5_H_6_N_3_O_2_)[ReO_4_]·H_2_O
*M* *_r_* = 408.34
Monoclinic, 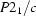

*a* = 9.6914 (3) Å
*b* = 9.1357 (3) Å
*c* = 13.4636 (4) Å
β = 120.120 (2)°
*V* = 1031.08 (6) Å^3^
*Z* = 4
Mo *K*α radiation
μ = 11.81 mm^−1^
*T* = 295 K
0.10 × 0.06 × 0.05 mm

#### Data collection

Bruker APEXII diffractometer
Absorption correction: multi-scan (*SADABS*; Sheldrick, 1996 ▶) *T*_min_ = 0.33, *T*_max_ = 0.55
30757 measured reflections
2374 independent reflections
2175 reflections with *I* > 2σ(*I*)
*R*_int_ = 0.030

#### Refinement

*R*[*F* ^2^ > 2σ(*F* ^2^)] = 0.015
*wR*(*F* ^2^) = 0.033
*S* = 1.05
2374 reflections
158 parameters
5 restraints
H atoms treated by a mixture of independent and constrained refinement
Δρ_max_ = 0.88 e Å^−3^
Δρ_min_ = −0.72 e Å^−3^

### 

Data collection: *APEX2* (Bruker, 2004 ▶); cell refinement: *SAINT* (Bruker, 2004 ▶); data reduction: *SAINT*; program(s) used to solve structure: *SHELXS97* (Sheldrick, 2008 ▶); program(s) used to refine structure: *SHELXL97* (Sheldrick, 2008 ▶); molecular graphics: *ORTEPII* (Johnson, 1976 ▶); software used to prepare material for publication: *SHELXL97* and *PLATON* (Spek, 2009).

## Supplementary Material

Crystal structure: contains datablocks I, global. DOI: 10.1107/S1600536809029365/ez2177sup1.cif

Structure factors: contains datablocks I. DOI: 10.1107/S1600536809029365/ez2177Isup2.hkl

Additional supplementary materials:  crystallographic information; 3D view; checkCIF report

## Figures and Tables

**Table 1:** Hydrogen-bond geometry (Å, °)

*D*—H⋯*A*	*D*—H	H⋯*A*	*D*⋯*A*	*D*—H⋯*A*
N1—H1⋯O3	0.86	1.93	2.738 (3)	157
N1—H1⋯O6^i^	0.86	2.63	3.049 (4)	112
N2—H2*A*⋯O3	0.89 (3)	2.33 (3)	3.030 (4)	135 (3)
N2—H2*A*⋯O4^ii^	0.89 (3)	2.65 (3)	3.259 (4)	127 (3)
N2—H2*B*⋯O5	0.89 (3)	1.98 (3)	2.846 (3)	164 (3)
O3—H3*A*⋯O7^iii^	0.89 (3)	1.93 (3)	2.802 (4)	167 (3)
O3—H3*B*⋯O4^ii^	0.89 (3)	2.13 (3)	2.862 (4)	139 (3)

Symmetry codes: (i) 
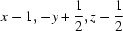
; (ii) 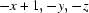; (iii) 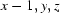.

**Fig. 1.**
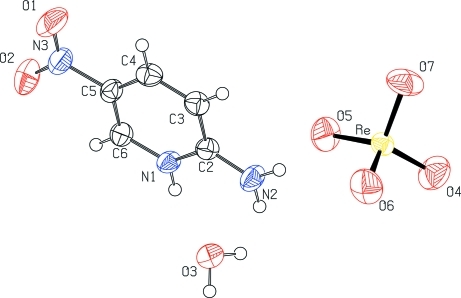
ORTEPII (Johnson, 1976) plot of the title compound. Displacement ellipsoids are drawn at the 50% level.

**Fig. 2.**
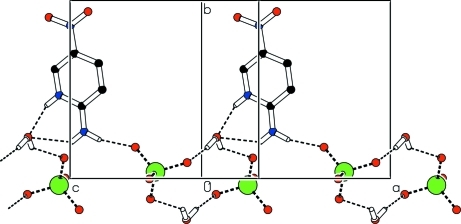
Zigzag chain running along the a axis. Small ring motifs made of a perrhenate anion and two water molecules are included in the chain. Hydrogen bonds are indicated with dashed lines.

## supplementary crystallographic information

### Experimental

Crystals of the title compound were obtained by slow evaporation from a water solution of analytical grade reagents on a 1:1 molar ratio. The reagents used were 2-amino-5-nitropyridine (99.5%) and a perrhenic acid solution (65–75% water 99.5%), both purchased from Aldrich.

### Refinement

The structure was solved by direct methods using *SHELXS97*. All H atoms were first located on a difference Fourier map as a check for data quality; those bonded to C and N aromatic atoms were then placed at idealized positions and refined as riding [C—H=0.93 Å, N—H=0.86 Å, *U*_iso_(H)=1.2*U*_eq_(C) and *U*_iso_(H)=1.2*U*_eq_(N)]. The coordinates of the amine H atoms were then refined only with the restraint that the N—H distance would be 0.89 Å within 0.2 Å. The positions of the H atoms belonging to the water molecule were also restrained so that the intramolecular O—H distance would be 0.89 Å and the H—H distance 1.40 Å, both within 0.02 Å, thus enforcing an acceptable geometry of the water molecule [*U*_iso_(H)=1.5*U*_eq_(O)].

Examination of the crystal structure with *PLATON* (Spek, 2009) showed that there are no solvent-accessible voids in the crystal lattice.

### Crystal data

**Table:**

(C_5_H_6_N_3_O_2_)[ReO_4_]·H_2_O	*F*(000) = 760
*M**_r_* = 408.34	*D*_x_ = 2.631 Mg m^−^^3^
Monoclinic, *P*2_1_/*c*	Mo *K*α radiation, λ = 0.71073 Å
Hall symbol: -P 2ybc	Cell parameters from 9920 reflections
*a* = 9.6914 (3) Å	θ = 2.4–32.3°
*b* = 9.1357 (3) Å	µ = 11.81 mm^−^^1^
*c* = 13.4636 (4) Å	*T* = 295 K
β = 120.120 (2)°	Prism, translucent colourless
*V* = 1031.08 (6) Å^3^	0.10 × 0.06 × 0.05 mm
*Z* = 4	

### Data collection

**Table:**

Bruker APEXII diffractometer	2374 independent reflections
Radiation source: fine-focus sealed tube	2175 reflections with *I* > 2σ(*I*)
graphite	*R*_int_ = 0.030
φ and ω scans	θ_max_ = 27.5°, θ_min_ = 2.4°
Absorption correction: multi-scan (*SADABS*; Sheldrick, 1996)	*h* = −12→12
*T*_min_ = 0.33, *T*_max_ = 0.55	*k* = −11→11
30757 measured reflections	*l* = −17→17

### Refinement

**Table:**

Refinement on *F*^2^	Secondary atom site location: difference Fourier map
Least-squares matrix: full	Hydrogen site location: inferred from neighbouring sites
*R*[*F*^2^ > 2σ(*F*^2^)] = 0.015	H atoms treated by a mixture of independent and constrained refinement
*wR*(*F*^2^) = 0.033	*w* = 1/[σ^2^(*F*_o_^2^) + (0.0122*P*)^2^ + 1.4451*P*] where *P* = (*F*_o_^2^ + 2*F*_c_^2^)/3
*S* = 1.05	(Δ/σ)_max_ = 0.001
2374 reflections	Δρ_max_ = 0.88 e Å^−^^3^
158 parameters	Δρ_min_ = −0.72 e Å^−^^3^
5 restraints	Extinction correction: *SHELXL97* (Sheldrick, 2008), Fc^*^=kFc[1+0.001xFc^2^λ^3^/sin(2θ)]^-1/4^
Primary atom site location: structure-invariant direct methods	Extinction coefficient: 0.00774 (16)

### Special details

**Table:**

Geometry. All e.s.d.'s (except the e.s.d. in the dihedral angle between two l.s. planes) are estimated using the full covariance matrix. The cell e.s.d.'s are taken into account individually in the estimation of e.s.d.'s in distances, angles and torsion angles; correlations between e.s.d.'s in cell parameters are only used when they are defined by crystal symmetry. An approximate (isotropic) treatment of cell e.s.d.'s is used for estimating e.s.d.'s involving l.s. planes.
Refinement. Refinement of *F*^2^ against ALL reflections. The weighted *R*-factor *wR* and goodness of fit *S* are based on *F*^2^, conventional *R*-factors *R* are based on *F*, with *F* set to zero for negative *F*^2^. The threshold expression of *F*^2^ > σ(*F*^2^) is used only for calculating *R*-factors(gt) *etc*. and is not relevant to the choice of reflections for refinement. *R*-factors based on *F*^2^ are statistically about twice as large as those based on *F*, and *R*- factors based on ALL data will be even larger.

### Fractional atomic coordinates and isotropic or equivalent isotropic displacement parameters (Å^2^)

**Table:**

	*x*	*y*	*z*	*U*_iso_*/*U*_eq_	
N1	0.2958 (3)	0.4684 (3)	0.0719 (2)	0.0338 (5)	
H1	0.2145	0.4119	0.0493	0.041*	
C2	0.4404 (3)	0.4063 (3)	0.1067 (2)	0.0315 (6)	
N2	0.4512 (3)	0.2632 (3)	0.1047 (3)	0.0434 (6)	
H2A	0.367 (3)	0.204 (3)	0.080 (3)	0.052*	
H2B	0.543 (2)	0.219 (4)	0.124 (3)	0.052*	
C3	0.5736 (3)	0.4998 (3)	0.1440 (3)	0.0352 (6)	
H3	0.6741	0.4605	0.1684	0.042*	
C4	0.5549 (3)	0.6463 (3)	0.1442 (2)	0.0362 (6)	
H4	0.6420	0.7086	0.1688	0.043*	
C5	0.4020 (3)	0.7029 (3)	0.1067 (2)	0.0332 (6)	
N3	0.3785 (4)	0.8592 (3)	0.1068 (2)	0.0437 (6)	
O1	0.4946 (4)	0.9377 (3)	0.1387 (3)	0.0717 (9)	
O2	0.2459 (3)	0.9055 (3)	0.0781 (3)	0.0632 (7)	
C6	0.2737 (3)	0.6133 (3)	0.0712 (2)	0.0336 (6)	
H6	0.1728	0.6517	0.0469	0.040*	
O3	0.1012 (3)	0.2314 (2)	0.0301 (2)	0.0538 (6)	
H3A	0.083 (5)	0.174 (3)	0.076 (2)	0.081*	
H3B	0.079 (5)	0.174 (3)	−0.030 (2)	0.081*	
Re	0.905465 (12)	0.037384 (13)	0.215124 (10)	0.03195 (6)	
O4	0.8222 (3)	−0.1153 (3)	0.1338 (2)	0.0680 (7)	
O5	0.7677 (3)	0.1758 (3)	0.1707 (3)	0.0632 (7)	
O6	0.9713 (4)	−0.0024 (3)	0.3551 (2)	0.0674 (8)	
O7	1.0618 (3)	0.0893 (3)	0.1987 (3)	0.0664 (8)	

### Atomic displacement parameters (Å^2^)

**Table:**

	*U*^11^	*U*^22^	*U*^33^	*U*^12^	*U*^13^	*U*^23^
N1	0.0264 (11)	0.0290 (12)	0.0452 (15)	−0.0007 (9)	0.0174 (11)	0.0031 (11)
C2	0.0289 (12)	0.0319 (14)	0.0329 (15)	0.0032 (11)	0.0150 (11)	0.0054 (12)
N2	0.0375 (13)	0.0281 (13)	0.0627 (18)	0.0052 (11)	0.0237 (14)	0.0057 (13)
C3	0.0262 (13)	0.0400 (16)	0.0369 (16)	0.0004 (11)	0.0142 (12)	0.0010 (13)
C4	0.0312 (13)	0.0411 (17)	0.0341 (16)	−0.0090 (12)	0.0147 (12)	−0.0035 (13)
C5	0.0417 (15)	0.0277 (14)	0.0296 (15)	0.0020 (12)	0.0175 (12)	0.0023 (12)
N3	0.0597 (17)	0.0299 (13)	0.0415 (15)	0.0009 (12)	0.0254 (13)	0.0003 (11)
O1	0.080 (2)	0.0345 (13)	0.108 (3)	−0.0181 (13)	0.0530 (19)	−0.0083 (14)
O2	0.0674 (17)	0.0398 (14)	0.0761 (19)	0.0152 (13)	0.0313 (15)	0.0003 (13)
C6	0.0320 (13)	0.0329 (15)	0.0351 (15)	0.0054 (12)	0.0161 (12)	0.0046 (13)
O3	0.0643 (16)	0.0382 (13)	0.0750 (19)	−0.0090 (12)	0.0471 (15)	−0.0043 (13)
Re	0.02646 (7)	0.03566 (8)	0.03418 (8)	0.00296 (4)	0.01554 (5)	0.00215 (5)
O4	0.0773 (19)	0.0537 (16)	0.0667 (18)	−0.0151 (14)	0.0315 (15)	−0.0156 (14)
O5	0.0447 (13)	0.0583 (16)	0.0843 (19)	0.0226 (12)	0.0307 (13)	0.0131 (15)
O6	0.085 (2)	0.0780 (19)	0.0394 (15)	0.0150 (16)	0.0313 (14)	0.0090 (13)
O7	0.0398 (13)	0.083 (2)	0.086 (2)	0.0057 (13)	0.0389 (14)	0.0225 (17)

### Geometric parameters (Å, °)

**Table:**

N1—C6	1.341 (4)	C5—C6	1.360 (4)
N1—C2	1.358 (3)	C5—N3	1.446 (4)
N1—H1	0.8600	N3—O1	1.216 (4)
C2—N2	1.313 (4)	N3—O2	1.217 (4)
C2—C3	1.413 (4)	C6—H6	0.9300
N2—H2A	0.89 (3)	O3—H3A	0.89 (3)
N2—H2B	0.89 (3)	O3—H3B	0.89 (3)
C3—C4	1.351 (4)	Re—O6	1.698 (3)
C3—H3	0.9300	Re—O7	1.704 (2)
C4—C5	1.402 (4)	Re—O4	1.705 (3)
C4—H4	0.9300	Re—O5	1.714 (2)
			
C6—N1—C2	123.2 (2)	C6—C5—N3	118.6 (3)
C6—N1—H1	118.4	C4—C5—N3	120.1 (3)
C2—N1—H1	118.4	O1—N3—O2	123.3 (3)
N2—C2—N1	119.2 (3)	O1—N3—C5	117.7 (3)
N2—C2—C3	122.8 (3)	O2—N3—C5	119.0 (3)
N1—C2—C3	118.0 (3)	N1—C6—C5	118.5 (2)
C2—N2—H2A	123 (2)	N1—C6—H6	120.7
C2—N2—H2B	122 (2)	C5—C6—H6	120.7
H2A—N2—H2B	115 (3)	H3A—O3—H3B	104 (3)
C4—C3—C2	120.0 (3)	O6—Re—O7	109.89 (15)
C4—C3—H3	120.0	O6—Re—O4	109.31 (15)
C2—C3—H3	120.0	O7—Re—O4	108.01 (15)
C3—C4—C5	118.9 (3)	O6—Re—O5	109.77 (14)
C3—C4—H4	120.6	O7—Re—O5	109.69 (13)
C5—C4—H4	120.6	O4—Re—O5	110.15 (14)
C6—C5—C4	121.3 (3)		
			
C6—N1—C2—N2	179.9 (3)	C4—C5—N3—O1	1.0 (4)
C6—N1—C2—C3	0.1 (4)	C6—C5—N3—O2	1.9 (4)
N2—C2—C3—C4	−179.9 (3)	C4—C5—N3—O2	−177.2 (3)
N1—C2—C3—C4	−0.1 (4)	C2—N1—C6—C5	0.1 (4)
C2—C3—C4—C5	−0.2 (4)	C4—C5—C6—N1	−0.4 (4)
C3—C4—C5—C6	0.4 (4)	N3—C5—C6—N1	−179.5 (3)
C3—C4—C5—N3	179.5 (3)	N1—C2—N2—H2A	1(3)
C6—C5—N3—O1	−179.9 (3)	C3—C2—N2—H2B	−3(3)

### Hydrogen-bond geometry (Å, °)

**Table:**

*D*—H···*A*	*D*—H	H···*A*	*D*···*A*	*D*—H···*A*
N1—H1···O3	0.86	1.93	2.738 (3)	157
N1—H1···O6^i^	0.86	2.63	3.049 (4)	112
N2—H2A···O3	0.89 (3)	2.33 (3)	3.030 (4)	135 (3)
N2—H2A···O4^ii^	0.89 (3)	2.65 (3)	3.259 (4)	127 (3)
N2—H2B···O5	0.89 (3)	1.98 (3)	2.846 (3)	164 (3)
O3—H3A···O7^iii^	0.89 (3)	1.93 (3)	2.802 (4)	167 (3)
O3—H3B···O4^ii^	0.89 (3)	2.13 (3)	2.862 (4)	139 (3)

Symmetry codes: (i) *x*−1, −*y*+1/2, *z*−1/2; (ii) −*x*+1, −*y*, −*z*; (iii) *x*−1, *y*, *z*.
